# Association Between Early Return to School Following Acute Concussion and Symptom Burden at 2 Weeks Postinjury

**DOI:** 10.1001/jamanetworkopen.2022.51839

**Published:** 2023-01-20

**Authors:** Christopher G. Vaughan, Andrée-Anne Ledoux, Maegan D. Sady, Ken Tang, Keith Owen Yeates, Gurinder Sangha, Martin H. Osmond, Stephen B. Freedman, Jocelyn Gravel, Isabelle Gagnon, William Craig, Emma Burns, Kathy Boutis, Darcy Beer, Gerard Gioia, Roger Zemek

**Affiliations:** 1 Division of Neuropsychology, Children’s National Hospital, Washington, DC; 2 Children’s Hospital of Eastern Ontario Research Institute, Ottawa, Ontario, Canada; 3 Department of Cellular and Molecular Medicine, University of Ottawa, Ottawa, Ontario, Canada; 4 Independent Statistical Consultant, Richmond, British Columbia, Canada; 5 Department of Psychology, Hotchkiss Brain Institute, and Alberta Children’s Hospital Research Institute, University of Calgary, Calgary, Alberta, Canada; 6 Department of Pediatrics, Children’s Hospital of Western Ontario, Western University, London, Ontario, Canada; 7 Department of Pediatrics, Children’s Hospital of Eastern Ontario, University of Ottawa, Ottawa, Ontario, Canada; 8 Department of Pediatrics, Alberta Children’s Hospital, Alberta Children’s Hospital Research Institute, University of Calgary, Calgary, Alberta, Canada; 9 Department of Pediatric Emergency Medicine, CHU Sainte-Justine, Université de Montréal, Montréal, Quebec, Canada; 10 Division of Pediatric Emergency Medicine, Department of Pediatrics, Montreal Children’s Hospital, McGill University Health Center, Montreal, Quebec, Canada; 11 School of Physical and Occupational Therapy, Faculty of Medicine and Health Sciences, McGill University, Montreal, Quebec, Canada; 12 Department of Pediatrics, Stollery Children’s Hospital, Edmonton, Alberta, Canada; 13 Department of Emergency Medicine, IWK Health, Dalhousie University, Halifax, Nova Scotia, Canada; 14 Hospital for Sick Children, University of Toronto, Toronto, Ontario, Canada; 15 Department of Pediatrics, Winnipeg Children’s Hospital, Winnipeg, Manitoba, Canada

## Abstract

**Importance:**

Determining how the timing of return to school is related to later symptom burden is important for early postinjury management recommendations.

**Objective:**

To examine the typical time to return to school after a concussion and evaluate whether an earlier return to school is associated with symptom burden 14 days postinjury.

**Design, Setting, and Participants:**

Planned secondary analysis of a prospective, multicenter observational cohort study from August 2013 to September 2014. Participants aged 5 to 18 years with an acute (<48 hours) concussion were recruited from 9 Canadian pediatric emergency departments in the Pediatric Emergency Research Canada Network.

**Exposure:**

The independent variable was the number of days of school missed. Missing fewer than 3 days after concussion was defined as an early return to school.

**Main Outcomes and Measures:**

The primary outcome was symptom burden at 14 days, measured with the Post-Concussion Symptom Inventory (PCSI). Symptom burden was defined as symptoms status at 14 days minus preinjury symptoms. Propensity score analyses applying inverse probability of treatment weighting were performed to estimate the relationship between the timing of return to school and symptom burden.

**Results:**

This cohort study examined data for 1630 children (mean age [SD] 11.8 [3.4]; 624 [38%] female). Of these children, 875 (53.7%) were classified as having an early return to school. The mean (SD) number of days missed increased across age groups (5-7 years, 2.61 [5.2]; 8-12 years, 3.26 [4.9]; 13-18 years, 4.71 [6.1]). An early return to school was associated with a lower symptom burden 14 days postinjury in the 8 to 12-year and 13 to 18-year age groups, but not in the 5 to 7-year age group. The association between early return and lower symptom burden was stronger in individuals with a higher symptom burden at the time of injury, except those aged 5 to 7 years.

**Conclusions and Relevance:**

In this cohort study of youth aged 5 to 18 years, these results supported the growing belief that prolonged absences from school and other life activities after a concussion may be detrimental to recovery. An early return to school may be associated with a lower symptom burden and, ultimately, faster recovery.

## Key Points

**Question:**

Is the timing of return to school after concussion associated with symptom burden at 14 days postinjury?

**Findings:**

In this cohort study, analysis of data collected from a multicenter sample of youth aged 5 to 18 years with concussion showed that older youth missed more days of school, on average, than younger youth. An early return to school was associated with a lower symptom burden at 14 days postinjury in the 8- to 12-year age group and 13- to 18-year age group.

**Meaning:**

These findings suggest that prolonged absence from school after a concussion is associated with a greater symptom burden and may be detrimental to recovery.

## Teaser

This cohort study examines the typical time to return to school after a concussion and evaluates whether an earlier return to school is associated with symptom burden at 14 days postinjury at 9 pediatric emergency departments in Canada.

## Introduction

A student’s return to school (RTS) after a concussion is a priority issue in recent consensus statements, living guidelines, and evidence-based reviews.^1,2,3^ Most guidance recommends 24 to 48 hours of physical and cognitive rest, followed by a gradual RTS with support and accommodations.^1,4^ However, little empirical evidence is available to inform clinical decisions about the length of school absence. Recommendations tend to be based on weaker evidence, such as consensus and usual practice.^1,4^

Several studies have attempted to characterize the distribution of when children return to school after concussion—72% will miss at least 1 day,^5^ and missing 2 to 5 days may be common.^3^ School absence has been used as a proxy for postconcussion cognitive rest. Thomas et al^6^ found that greater cognitive rest, which included more time away from school, was associated with a longer recovery. Nonetheless, early RTS also has the potential to exacerbate symptoms and delay recovery. In a study of 42 student-athletes, more time at school was associated with higher symptom levels.^7^ Evidence demonstrates that early return to physical activity is beneficial for concussion recovery,^4,8,9,10^ but prospective studies of associations between RTS timing and symptom recovery, with adequate power to consider confounders, such as early symptom burden and injury characteristics, are lacking.

The aims of this study were to (1) characterize the duration of school absence in a large, prospective, multicenter pediatric cohort enrolled within 48 hours of concussion and (2) examine associations between RTS timing (early vs late RTS) and symptom burden at 14 days (ie, 2 weeks) postinjury. Based on recent literature, including current guidelines,^1,4^ we hypothesized that most students would initiate RTS after less than 1 week. We also hypothesized that an early RTS would be associated with a lower overall symptom burden 14 days later.

## Methods

### Study Design

This analysis used data from the Predicting Persistent Postconcussion Problems in Pediatrics (5P) study, a prospective cohort study of youth with a concussion, conducted between August 2013 and June 2015 in 9 pediatric emergency departments (EDs) of the Pediatric Emergency Research Canada (PERC).^11,12,13^ The study was approved by all 9 research ethics committees at the enrolling institutions. Informed consent was obtained from parents and participants. If youth participants were unable to give consent, assent was required. This study adhered to Strengthening the Reporting of Observational Studies in Epidemiology (STROBE) reporting guideline for cohort studies.

### Study Population

The original SP cohort comprised 3063 youth aged 5.00 to 17.99 years diagnosed with concussion based on the 2012 Zurich Consensus Statement criteria.^14^ Cohort inclusion and exclusion criteria have previously been published. In summary, eligible participants presented at a site ED with symptoms of a concussion sustained within 48 hours. Patients who did not have an injury based on trauma, who had a Glasgow Coma Score of 13 or less or abnormal findings on brain CT or MRI, or who required hospital admission for multisystemic injury or neurosurgical intervention were excluded. Youth were also excluded if they had severe developmental delay, limited communication abilities, intoxication, or had previously enrolled in the same study.

For the present analysis, participants also required a valid and specific RTS date. Youth whose injury occurred during typical periods of school closure (ie, summer vacation, Christmas or winter holiday, and March break) or who had a prolonged absence (ie, >20 days) that extended through 1 of those breaks were excluded.

### Study Protocol

Following consent, trained research assistants collected information from the patient or family and medical chart using a standardized protocol within the EDs and via the Research Electronic Data Capture (REDCap) system.^15,16^ Demographic, developmental and psychiatric data, and medical histories, including the presence and timing of prior concussions and pre-injury headaches, were gathered. The Acute Concussion Evaluation (ACE)^17^ was used in the ED to capture the presence or absence of symptoms. The developmentally appropriate Post-Concussion Symptom Inventory (PCSI) provided initial and retrospective pre-injury patient reports of symptom severity.

### Follow-up Procedures

At 7, 14, and 28 days postinjury, participants were contacted via web survey or telephone and asked to provide information, including their RTS date and current symptom burden. Parents responded on behalf of participants aged 5 to 7 years, and responses were collected directly from participants aged 8 to 17 years.

### Primary Outcome Measure

The primary outcome was symptom burden measured by the PCSI score at 14 days postinjury. This time point was selected to obtain the most proximal symptom assessment after likely RTS. Symptom burden was a delta score of the current symptom rating minus the retrospectively reported pre-injury level of the symptom. Retrospectively adjusted scores that fell in the negative range were considered to be zero. Unique versions of the PCSI administered to the 3 age groups contained 13, 17, and 20 items, with each item rated on scales of 0 to 2, 0 to 2, and 0 to 6,^18^ respectively. The possible PCSI score ranges for these versions were 0 to 26, 0 to 34, and 0 to 120, respectively.

### Main Estimator

The primary variable assessed—the number of school days missed—was calculated excluding weekends. The modal number of days missed for all 3 age groups was 2, so early RTS was missing 0 to 2 days, and late RTS was missing 3 or more days. Of further interest was whether the association between the number of missed school days and symptom burden at day 14 varied depending on symptom burden at the initial study presentation.

### Statistical Analysis

To address aim 1, descriptive statistics (mean, SD, median, quartiles, minimum, and maximum) were calculated to summarize the distributions of days missed, by age group. To address aim 2, propensity score analyses^19^ applying inverse probability of treatment weighting (IPTW) were performed to estimate the relationship between early or late RTS and the PCSI delta score at day 14. A propensity score (PS) helps to level the playing field by accounting for the likelihood of an outcome based on multiple predetermined factors. This single PS is used, instead of the set of pre-existing factors, to examine the additional association between the variable of interest and the outcome. Three sets of PS analyses were performed, 1 for each age group.

The first step was to derive PS models by fitting logistic regressions to estimate the probability of early RTS, given all available variables determined a priori from clinical experience, previous research,^12^ and plausible relationship with our main estimator and/or study outcome. Parameter-to-event ratios of no worse than about 5:1 were required to avoid overparameterization in these PS models. For the 8 to 12 year and 13 to 18 year age groups, these variables were: study site, age, sex, the maximum duration of prior concussion, the time between concussion and triage, prior headache treatment, personal migraine history, family migraine history, learning disability, attention-deficit hyperactivity disorder, other developmental disorders, anxiety, postinjury seizure, early signs on the Acute Concussion Evaluation (ie, appears dazed or stunned, confused about events, answers questions slowly, repeats questions, forgetful), 5 mechanisms of injury (ie, sports or recreational play, nonsports or fall, motor vehicle collision, assault, other), BESS tandem stance errors (0-10), anterograde amnesia, 4 standardized assessment of concussion (SAC) subscores (orientation, immediate memory, concentration, delayed recall), normalized SAC total score, all 20 parent-reported PCSI pre-injury item scores, frontal head impact, parietal head impact, temporal head impact, occipital head impact, face impact, mandible injury, and day of the week when injury occurred (7 levels, to include the possible recovery benefit of being injured before or during a weekend). For 5- to 7-year-olds, fewer events were observed; therefore, a subset of variables was included sex, maximum duration of prior concussion, time between concussion and triage, personal migraine history, injury mechanism, BESS tandem stance test errors (0-10), SAC normalized total score, 4 parent-reported PCSI subscale scores (physical, emotional, fatigue, cognitive) in lieu of the 20 specific items, and day of initial injury. In all 3 PS models, we applied restricted cubic splines with 3 knots to key continuous variables (age, time between injury and triage, SAC total) to allow for nonlinearity. Listwise deletion was applied. The c-stat was used to gauge the models’ ability to discriminate between early vs late RTS.

The second step involved applying inverse probability of treatment weighting (IPTW) to estimated PS from the first step and then assessing covariate balance and degree of residual imbalances. The IPTW is the reciprocal of the probability of an early RTS for patients who reported an early RTS, and the probability of a late RTS for patients who reported a late RTS. Balance diagnostics included assessments of density plots, as well as changes in absolute standardized mean difference (SMD) before and after the application of IPTW for each individual variable in the PS model (unweighted vs weighted samples). In the context of PS modeling, an SMD of less than 0.1 has been regarded as a balanced covariate.^20,21^

The third step involved fitting IPTW-weighted linear regression models to quantify the relationship between RTS and the day 14 PCSI delta score, while incorporating IPTW. The independent variables consisted, first, of an interaction between RTS timing and the total ED visit PCSI delta score and, second, a double adjustment of covariates by reintroducing individual variables with SMD greater than 0.1 after the IPTW procedure to further adjust for residual imbalances of covariates across the early vs late RTS groups. Restricted cubic splines with 4 knots were applied to all continuous independent variables (eg, age, total ED visit PCSI score) to allow for nonlinearity. To detail the association between RTS timing and outcome, postmodel fit contrasts (ie, early vs late RTS) at selected baseline PCSI quantiles (ie, p10, p25, p50, p75, p90) were estimated, along with 95% CIs and associated *P* values. An overall association was estimated by a weighted averaging of all possible contrasts (weighted by the number of contributing observations per contrast). The α was set at .05, and *R* version 3.5.2 (R Project for Statistical Computing) was used to run statistical analyses.^22^ Statistical analysis was completed between May 2017 and October 2019.

## Results

**Figure 1.**
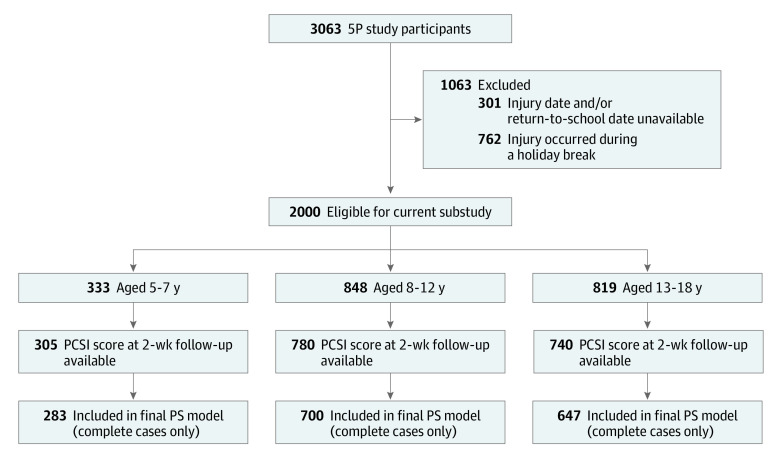
Participant Enrollment Flowchart Illustrates the number of eligible observations from the original 5P (Predicting and Preventing Postconcussive Problems in Pediatrics) study participants after applying exclusion criteria for the current substudy, the number of observations with Post-Concussion Symptom Inventory (PCSI) score at 2 weeks (primary study outcome), and number of observations included in the final propensity score models (ie, have complete data on primary outcome and all model covariates), stratified by age groups (5-7, 8-12, and 13-18 year olds).

The timing of the injury and known RTS date criteria led to the initial retention of 2000 (65.3%) of the original 5P study sample (Figure 1). Our analyses included a total of 1630 participants (624 [38%] female; mean [SD] age, 11.8 [3.4] years), across 3 age groups (5.0-7.9 years, 283 [17.4%]; 8.0-12.9 years, 700 [42.9%]; and 13.0-17.9 years, 647 [39.7%]) had complete data, including symptom status at day 14, and were included in our analysis. Among 5- to 7-year-olds, no differences in the PS analysis variables emerged between those included in the analyses and those excluded based on the timing of the injury and known RTS date. Among 8- to 12-year-olds, those excluded were more likely to have received previous headache treatment; less likely to have early signs of appearing confused or forgetful; less likely to have an early symptom of forgetfulness; and less likely to have sustained the concussion from an impact in parietal or temporal regions of the head. Among 13- to 18-year-olds, those excluded were slightly older, more likely to have received prior headache treatment or to have a personal migraine history, and more likely to have an early symptom of sadness or nervousness (eTables 1-3 in Supplement 1). Table 1 summarizes characteristics by age group.

**Table 1.** Descriptive Statistics by Age Group

Variable	Age Group, No. (%)		
	5-7 y (No. = 283)	8-12 y (No. = 700)	13-18 y (No. = 647)
Age, mean (SD), y	6.61 (0.84)	10.70 (1.41)	15.08 (1.24)
Sex			
Male	180 (63.6)	473 (67.6)	353 (54.6)
Female	103 (36.4)	227 (32.4)	294 (45.4)
Maximum symptom duration from previous concussion(s), wk			
Never had concussion	249 (88.0)	580 (82.9)	425 (65.7)
<1	25 (8.8)	68 (9.7)	76 (11.7)
1-2	4 (1.4)	19 (2.7)	51 (7.9)
3-4	2 (0.7)	15 (2.1)	40 (6.2)
5-8	0 (0.0)	6 (0.9)	19 (2.9)
>8	3 (1.1)	12 (1.7)	36 (5.6)
Time between head injury and triage, mean (SD), h	6.36 (9.02)	7.90 (11.08)	11.00 (13.20)
Prior treatment for headache	24 (8.5)	97 (13.9)	124 (19.2)
Personal history of migraine	17 (6.0)	70 (10.0)	106 (16.4)
Family history of migraine			
No	132 (46.6)	346 (49.4)	337 (52.1)
Yes	142 (50.2)	343 (49.0)	296 (45.7)
Unknown	9 (3.2)	11 (1.6)	14 (2.2)
History of learning disability	11 (3.9)	49 (7.0)	53 (8.2)
History of attention deficit disorder	11 (3.9)	64 (9.1)	56 (8.7)
History of other developmental disorders	10 (3.6)^a^	29 (4.1)	21 (3.2)
History of anxiety	11 (3.9)	44 (6.3)	73 (11.3)
Seizure following injury	8 (2.8)^b^	13 (1.9)	9 (1.4)
Mechanism of injury			
Sports or recreation	139 (49.1)	477 (68.1)	520 (80.4)
Nonsport or fall	138 (48.8)	208 (29.7)	103 (15.9)
Motor vehicle collision	3 (1.1)	7 (1.0)	12 (1.9)
Assault	3 (1.1)	8 (1.1)	11 (1.7)
Other	0	0	1 (0.2)
BESS tandem stance No. of errors, mean (SD)	4.78 (3.70)	3.76 (3.56)	3.63 (3.78)
Amnesia following injury	100 (35.3)	223 (31.9)	203 (31.4)
SAC, mean (SD), raw score			
Orientation	2.31 (1.47)^a^	3.60 (0.72)	3.76 (0.51)
Immediate memory	11.10 (3.04)	13.47 (1.68)	13.79 (1.60)
Concentration	2.30 (1.27)^c^	3.98 (1.19)	4.50 (1.20)
Delayed recall	3.42 (1.68)	4.09 (1.15)	4.07 (1.19)
Total score, z-score	−1.36 (1.97)	0.05 (1.19)	−0.34 (1.55)
PCSI summed score at ED visit, mean (SD), Δ score	6.87 (4.33)	11.10 (5.99)	34.82 (21.32)

Abbreviations: BESS, balance error scoring system; ED, emergency department; PCSI, Post-Concussion Symptom Inventory; SAC, standardized assessment of concussion.

^a^ No. = 281.

^b^ No. = 282.

^c^ No. = 279.

### Number of School Days Missed

**Figure 2.**
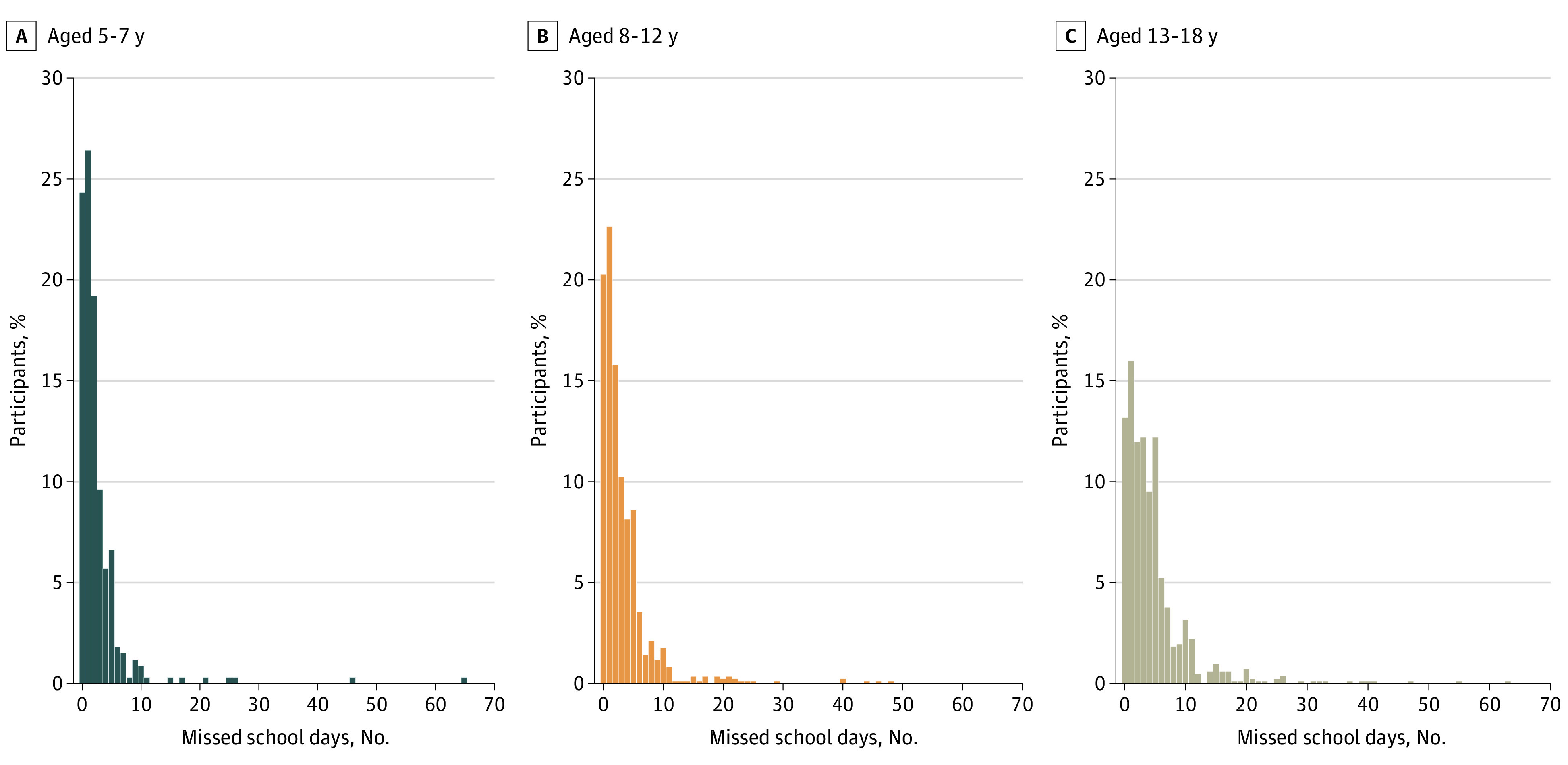
Number of Missed School Days by Age Group Missed school days by age group. Distribution of the number of missed school days following concussion is presented for each of 3 age groups (5-7, 8-12, and 13-18 year olds). The y-axis depicts the percentage of participants within each specific age group.

Across the total sample, the mean (SD) number of school days missed was 3.74 (5.5), excluding weekends. Older children had a higher number of days missed (5-7 years: mean [SD], 2.61 [5.2]; 8-12 years: mean [SD], 3.26 (4.9) years; 13-18 years: mean [SD], 4.71 [6.1]) (Figure 2). A total of 875 participants (53.7%) had an early RTS of 2 or fewer missed days, while 755 (46.3%) missed 3 or more days (late RTS). Late RTS occurred most often among the 13 to 17 years age group (383 of 647 [59.2%]), followed by the 8 to 12 years (285 of 700 [40.7%]) and 5 to 7 (87 of 283 [30.7%]) age groups.

### Propensity Score Matched Analysis

The PS matching models demonstrated good overlap with a c-statistic of 0.738 for 5- to 7-year-olds, 0.773 for 8- to 12-year-olds, and 0.769 for 13- to 18-year-olds (eFigures 1 and 2 in Supplement 1). Significant overall associations were found between early RTS and lower mean symptoms levels at day 14 across the 8- to 12-year age group (early vs late RTS estimate, −1.668; 95% CI, −2.339 to −0.997; *P* < .001) and 13- to 18-age groups (early vs late RTS estimate, −3.145; 95% CI, −5.247 to −1.043; *P* = .003) (eFigure 3 in Supplement 1). Given differences in scaling of the PCSI across these 2 age groups, for the purpose of comparability across age groups, standardized mean differences (divided by the respective age group SDs) show a standardized effect of −0.35 in the 8- to 12-year age group and −0.21 in the 13- to 18- year age group. There was no association between early RTS and fewer symptoms in the 5- to 7-year age group (early vs late RTS estimate = −0.71; 95% CI, −1.43-0.01; *P* = .05).

**Figure 3.**
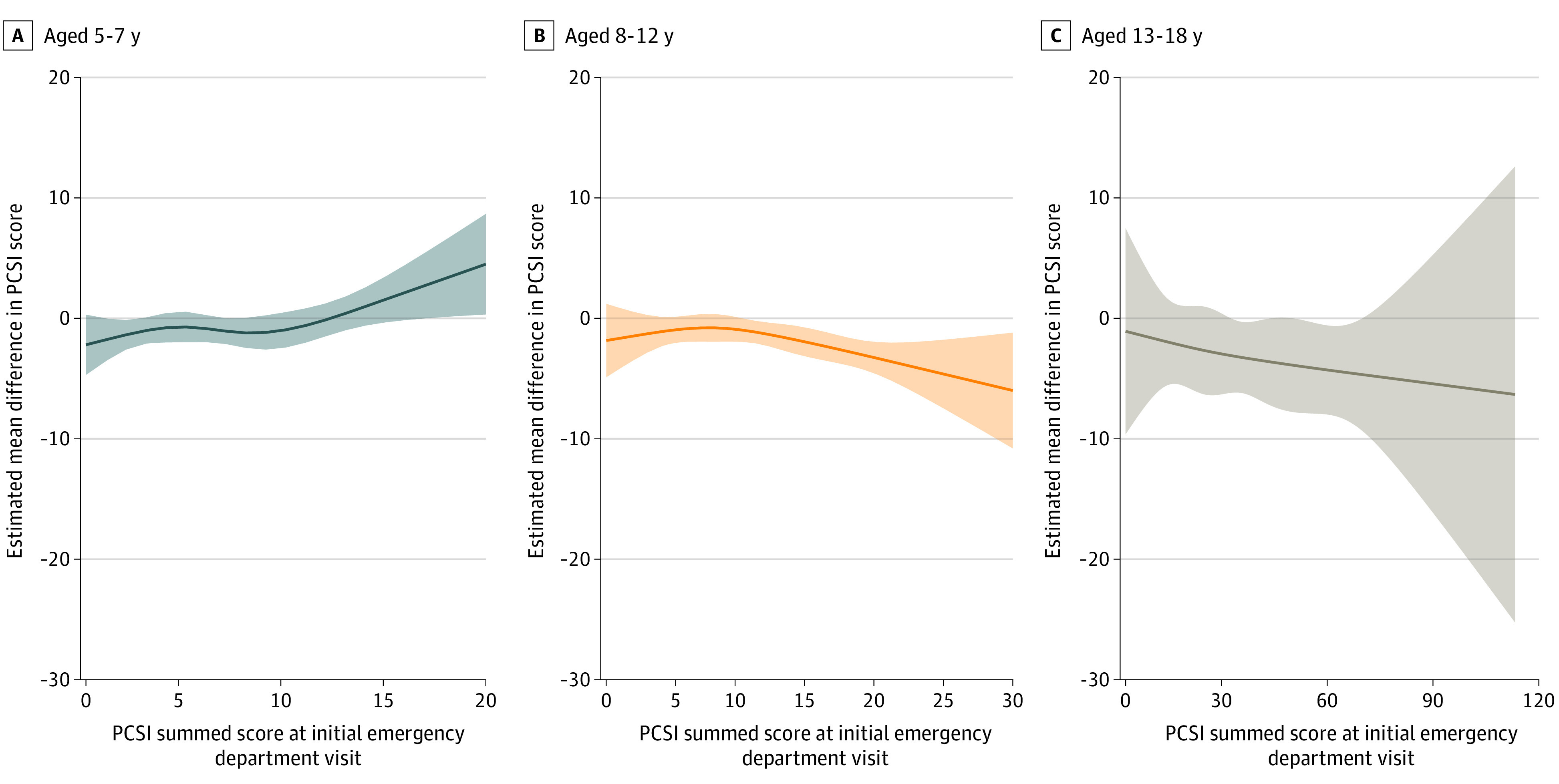
Association Between Early School Return and Symptom Level at 2 Weeks Postinjury by Initial Symptom Quintiles Estimated association of early return-to-school on the primary study outcome at different baseline symptom severity. The x-axis represents baseline symptom severity quantified by the Post-Concussion Symptom Inventory (PCSI) summed score at the initial emergency department visit (baseline). The y-axis represents the estimated mean difference in PCSI summed score at 2 weeks between early and late return-to-school groups. A positive value indicates early return-to-school is associated with an increase in PCSI summed score at 2 weeks (more symptoms). A negative value indicates early return-to-school is associated with an decrease in PCSI summed score at 2 weeks (fewer symptoms). These estimates are derived from the final propensity score models fitted separately for each of 3 age groups (5-7, 8-12, and 13-18 year olds), where an interaction between timing of return-to-school and PCSI summed score at the initial emergency department visit was specified.

To better understand the nature of the association between early RTS and decreased symptom burden at day 14, quantiles were established based on initial symptom ratings (day of injury) (Table 2, Figure 3). For 5- to 7-year-olds, the association was stronger among those with lower initial symptom levels (initial PCSI at p10: early vs late RTS estimate, −1.37; 95% CI, −2.60 to −0.15). The pattern changed among 8- to 12-year-olds, with a stronger association among those with higher initial symptom levels (initial PCSI at p75: early vs late RTS estimate = −2.04; 95% CI, −3.24 to −0.84; initial PCSI at p90: early vs late RTS estimate, −3.06; 95% CI, −4.26 to −1.85). Similarly, for 13- to 18-year-olds, a stronger association was found among those with higher initial symptom levels (baseline PCSI at p50: early vs late RTS estimate, −3.10; 95% CI, −6.18 to −0.03; baseline PCSI at p75: early vs late RTS estimate, −3.85; 95% CI, −7.73 to 0.03), and baseline PCSI at p90: early vs late RTS estimate, −4.48; 95% CI, −8.37 to −0.58). In summary, children and youth aged 8 to 18 years with higher initial symptoms who returned to school early had a lower symptom burden at day 14, even when their likelihood to return to school early and other known factors were considered.

**Table 2.** Effect of Early vs Late RTS by Quantiles of Initial Symptom for Each Age Group^a^

Initial PCSI	Summed score^b^	Early vs late RTS, SMD (95% CI)	*P* value
**Age 5-7 years** ^c^			
Overall		−0.709 (−1.430 to 0.013)	.05
p10	2	−1.371 (−2.595 to −0.146)	.03
p25	3	−1.020 (−2.102 to 0.063)	.07
p50	6	−0.852 (−1.973 to 0.269)	.14
p75	10	−0.955 (−2.421 to 0.512)	.20
p90	13	0.419 (−0.981 to 1.819)	.56
**Age 8-12 years** ^d^			
Overall		−1.668 (−2.339 to −0.997)	<.001
p10	3.9	−1.134 (−2.399 to 0.131)	.08
p25	6	−0.858 (−1.942 to 0.226)	.12
p50	10	−0.972 (−1.950 to 0.006)	.05
p75	15	−2.036 (−3.238 to −0.835)	.001
p90	19	−3.055 (−4.260 to −1.851)	<.001
**Age 13-18 years** ^e^			
Overall		−3.145 (−5.247 to −1.043)	.003
p10	10	−1.799 (−5.951 to 2.353)	.40
p25	19	−2.419 (−5.859 to 1.021)	.17
p50	31	−3.103 (−6.178 to −0.028)	.05
p75	48	−3.852 (−7.733 to 0.028)	.05
p90	64	−4.475 (−8.370 to −0.579)	.02

Abbreviations: PCSI, Post-Concussion Symptom Inventory; RTS, return to school; SMD, standardized mean difference.

^a^ The contrast for all groups was early vs late RTS.

^b^ The total score of each of the quantiles listed.

^c^ Additional model covariates (SMD > 0.1) for ages 5 to 7 years: site number, sex, maximum symptom duration from previous concussion(s), mechanism of injury, balance error scoring system tandem stance number of errors, and day of initial injury.

^d^ Additional model covariates (SMD > 0.1) for ages 8 to 12 years: day of initial injury.

^e^ Additional model covariates (SMD > 0.1) for ages 13 to 18 years: site and day of initial injury.

## Discussion

In this prospective cohort study, the mean number of school days missed due to concussion was 3 to 5 days. On average, younger children returned to school after concussion earlier than older children. The age difference suggests that what may be clinically considered early RTS for an adolescent may not be early RTS for a younger child. Based on a constant definition, regardless of age, for the purpose of the analyses, earlier RTS (ie, in 2 days or fewer) was associated with lower symptom levels at day 14 in children and youth aged 8 years or older. Surprisingly, earlier RTS was associated with lower symptom levels at day 14 among those with higher initial symptom. The results of this study support the possibility that an earlier RTS is associated with a lower symptom burden at 14 days postinjury and may directly or indirectly promote faster recovery.

Consistent with these findings, a descriptive case series suggested that patients who followed recommendations for a slower return to activity (physical and cognitive rest, including restriction from school activities and electronics) had a longer recovery period and greater symptom burden at 10 days postinjury, on average, than did those who did not follow these guidelines.^23^ An emergency department study conducted in the United States reported that school absences were longer (ie, median, 3.0 days) for the 20% of the sample with symptoms persisting beyond 1-month postinjury compared with those who recovered more quickly (ie, median, 1.5 days). However, the study did not account for symptom severity or possible injury severity.^5^

The finding that earlier RTS may be associated with a lower symptom burden at 14 days may be linked to the benefits of socialization, reduced stress from not missing too much school, maintaining or returning to a normal sleep-wake schedule, and returning to light-to-moderate physical activity (eg, gym class and recreational activities).^9^ Indeed, school absences for concussion or orthopedic injury can result in lower health-related quality of life (QOL), although school-related QOL may initially be worse after a concussion.^24^ Prolonged activity restriction after concussion is postulated to increase the risk for anxiety and depression and to have deleterious effects on overall physical health and symptom burden.^9,25,26^ School absence may also increase screentime, which a recent randomized control trial of teenagers with concussion identified as possibly being deleterious to recovery in the initial 48 hours.^27^

Further research should examine the importance of school-based concussion supports and resources, as they likely affect associations between RTS and symptoms. Prolonged absence may be detrimental to some students yet benefit others, depending on symptom profiles. Exercise and socialization could be embedded benefits of early RTS.

The factors and mechanisms behind the timing of RTS, the effect RTS has on symptom burden, and the length of recovery remain to be determined. This study offers evidence of the benefit of an earlier RTS in reducing symptom burden at 14 days, but the factors and reasons underlying this association remain unknown. Differences in injury, symptoms, and activity tolerance should be considered when providing individualized clinical guidance. These findings support current guidelines indicating that early RTS can benefit to physical and mental health. We recommend early RTS with accommodations as required.

A major strength of this study is the generalizability of the results. The large prospective cohort was assembled from 9 study sites. Participants presented with concussions caused by various injury mechanisms (not just sports), with equal representation of boys and girls.

### Limitations and Future Directions

This study had limitations. One limitation was that RTS timing was not randomly assigned prospectively, and thus, causality could not be determined. Sophisticated analytical methods were used to statistically balance the groups according to pre- and postinjury characteristics that could otherwise have led to self-selection for an earlier or later RTS based solely on injury severity or other factors. However, the result of injury severity on selection bias appears to be minimal. Several post hoc analyses found that individuals with a greater initial symptom burden (ie, students aged 8 and older) benefited most from early RTS compared with those with a milder initial symptom burden. Initial school absence is likely to be based on factors, such as symptom severity, early treatment recommendations, and prior experiences with a concussion. Upon returning to school, individuals likely received a variety of supports and accommodations, which was not measured and could have conceivably affected the early return or late return cohorts differently.

## Conclusions

In this cohort study, among children and youth aged 8 to 18 years with an acute concussion, RTS within 2 days of acute injury was associated with lower symptom burden at 14 days postinjury compared with later return. A randomized clinical trial is required to determine the best timing for RTS.

## Notes

Supplement 1. **eTable 1.** Descriptive Statistics of Key Variables Applied in Propensity Score Analysis for Each Age Group, Stratified by Early vs Late Return-to-School Ages 5 to 7 Years **eTable 2.** Descriptive Statistics of Key Variables Applied in Propensity Score Analysis for Each Age Group, Stratified by Early vs Late Return-to-School Ages 8 to 12 Years **eTable 3.** Descriptive Statistics of Key Variables Applied in Propensity Score Analysis for Each Age Group, Stratified by Early vs Late Return-to-School Ages 13 to 18 Years **eFigure 1. **Standardized Mean Differences Before and After Applying IPTW (Age 5-7 Years) **eFigure 2.** Standardized Mean Differences Before and After Applying IPTW (Age 8-12 Years and Age 13-18 Years) **eFigure 3.** PCSI Summed Score at 2 Week Follow-up (Primary Study Outcome), Stratified by Early of Late Return-to-School and Age Group

Supplement 2.
Nonauthor Collaborators

Supplement 3.
Data Sharing Statement
