# Supplementary material for: Association Between Early Return to School Following Acute Concussion and Symptom Burden at 2 Weeks Postinjury
Source: JAMA Netw Open. 2023 Jan 20;6(1):e2251839. doi: 10.1001/jamanetworkopen.2022.51839 (PMC9860528; doi:10.1001/jamanetworkopen.2022.51839)
Supplement: Supplement 1. — eTable 1. Descriptive Statistics of Key Variables Applied in Propensity Score Analysis for Each Age Group, Stratified by Early vs Late Return-to-School Ages 5 to 7 Years eTable 2. Descriptive Statistics of Key Variables Applied in Propensity Score Analysis for Each Age Group, Stratified by Early vs Late Return-to-School Ages 8 to 12 Years eTable 3. Descriptive Statistics of Key Variables Applied in Propensity Score Analysis for Each Age Group, Stratified by Early vs Late Return-to-School Ages 13 to 18 Years eFigure 1. Standardized Mean Differences Before and After Applying IPTW (Age 5-7 Years) eFigure 2. Standardized Mean Differences Before and After Applying IPTW (Age 8-12 Years and Age 13-18 Years) eFigure 3. PCSI Summed Score at 2 Week Follow-up (Primary Study Outcome), Stratified by Early of Late Return-to-School and Age Group [file jamanetwopen-e2251839-s001.pdf]

Supplemental Online Content

Vaughan CG, Ledoux AA, Sady MD, et al; PERC 5P Concussion Team. Association between early return to school following acute concussion and symptom burden at 2 weeks postinjury. *JAMA Network Open*. 2023;6(1):e2251839. doi:10.1001/jamanetworkopen.2022.51839

eTable 1. Descriptive Statistics of Key Variables Applied in Propensity Score Analysis for Each Age Group, Stratified by Early vs Late Return-to-School Ages 5 to 7 Years
eTable 2. Descriptive Statistics of Key Variables Applied in Propensity Score Analysis for Each Age Group, Stratified by Early vs Late Return-to-School Ages 8 to 12 Years
eTable 3. Descriptive Statistics of Key Variables Applied in Propensity Score Analysis for Each Age Group, Stratified by Early vs Late Return-to-School Ages 13 to 18 Years
eFigure 1. Standardized Mean Differences Before and After Applying IPTW (Age 5-7 Years)
eFigure 2. Standardized Mean Differences Before and After Applying IPTW (Age 8-12 Years and Age 13-18 Years)
eFigure 3. PCSI Summed Score at 2 Week Follow-up (Primary Study Outcome), Stratified by Early of Late Return-to-School and Age Group

This supplemental material has been provided by the authors to give readers additional information about their work.

© 2023 Vaughan CG et al. *JAMA Network Open.*

eTable 1. Descriptive statistics of Key Variables Applied in Propensity Score Analysis for Each Age Group, Stratified by Early vs Late Return-to-School for Participants Ages 5 to 7 Years

Variable	*N*	Early RTS: 0-2 missed school days (n=196) No. (%)	Late RTS: 3+ missed school days (n=87) No. (%)
**Age, mean (SD), years**	283	6.60 (0.84)	6.63 (0.84)
**Sex**	283		
Male		123 (62.8)	57 (65.5)
Female		73 (37.2)	30 (34.5)
**Maximum symptom duration from previous concussion(s) (weeks)**	283		
Never had concussion		173 (88.3)	76 (87.4)
<1 week		16 (8.2)	9 (10.3)
1-2 weeks		2 (1.0)	2 (2.3)
3-4 weeks		2 (1.0)	0 (0.0)
>8 weeks		3 (1.5)	0 (0.0)
**Personal history of migraine**	283	14 (7.1)	3 (3.4)
**Time between head injury and triage, mean (SD), hours**	283	6.19 (8.97)	6.74 (9.17)
**PCSI-P physical (0-6) at initial ED visit, mean (SD), delta score**	283	1.36 (1.01)	1.51 (1.07)
**PCSI-P emotional (0-6) at initial ED visit, mean (SD), delta score**	283	0.97 (1.10)	1.31 (1.36)
**PCSI-P fatigue (0-6) at initial ED visit, mean (SD), delta score**	283	2.49 (1.90)	2.65 (2.00)
**PCSI-P cognitive (0-6) at initial ED visit, mean (SD), delta score**	283	0.83 (1.14)	1.15 (1.36)
**SAC total, mean (SD), total (normalized)**	283	-1.27 (1.95)	-1.57 (2.03)
**Mechanism of Injury**	283		
Sports/Recreation		100 (51.0)	39 (44.8)
Non-sport/Fall		93 (47.4)	45 (51.7)
Motor vehicle collision		0 (0.0)	3 (3.4)
Assault		3 (1.5)	0 (0.0)
**BESS tandem stance, mean (SD), No. errors**	283	4.68 (3.67)	4.99 (3.78)
**Day of initial injury**	283		
Sunday		24 (12.2)	15 (17.2)
Monday		20 (10.2)	15 (17.2)
Tuesday		24 (12.2)	19 (21.8)
Wednesday		39 (19.9)	11 (12.6)
Thursday		38 (19.4)	16 (18.4)
Friday		26 (13.3)	8 (9.2)
Saturday		25 (12.8)	3 (3.4)
**PCSI-C score at ED visit, mean (SD), total**	283	6.74 (4.24)	7.15 (4.54)

© 2023 Vaughan CG et al. *JAMA Network Open.*

eTable 2. Descriptive statistics of Key Variables Applied in Propensity Score Analysis for Each Age Group, Stratified by Early vs Late Return-to-School for Participants Ages 8 to 12 Years

Variable	*N*	Early RTS: 0-2 missed school days (n=415) No. (%)	Late RTS: 3+ missed school days (n=285) No. (%)
**Age, mean (SD), years**	700	10.56 (1.39)	10.90 (1.42)
**Sex**	700		
Male		285 (68.7)	188 (66.0)
Female		130 (31.3)	97 (34.0)
**Maximum symptom duration from previous concussion(s) (weeks)**	700		
Never had concussion		354 (85.3)	226 (79.3)
<1 week		38 (9.2)	30 (10.5)
1-2 weeks		7 (1.7)	12 (4.2)
3-4 weeks		7 (1.7)	8 (2.8)
5-8 weeks		4 (1.0)	2 (0.7)
>8 weeks		5 (1.2)	7 (2.5)
**Time between head injury and triage, mean (SD), hours**	700	6.28 (9.14)	10.25 (13.09)
**Prior treatment for headache**	700	48 (11.6)	49 (17.2)
**Personal history of migraine**	700	36 (8.7)	34 (11.9)
**Family history of migraine**	700		
No		207 (49.9)	139 (48.8)
Yes		199 (48.0)	144 (50.5)
Unknown		9 (2.2)	2 (0.7)
**History of learning disability**	700	25 (6.0)	24 (8.4)
**History of attention deficit disorder**	700	32 (7.7)	32 (11.2)
**History of other developmental disorders**	700	15 (3.6)	14 (4.9)
**History of anxiety**	700	22 (5.3)	22 (7.7)
**Seizure following injury**	700	8 (1.9)	5 (1.8)
**ACE - Appears dazed and confused**	700	193 (46.5)	156 (54.7)
**ACE - Appears confused about events**	700	84 (20.2)	79 (27.7)
**ACE - Answers questions slowly**	700	153 (36.9)	134 (47.0)
**ACE - Repeats questions**	700	43 (10.4)	49 (17.2)
**ACE - Forgetful of recent information**	700	64 (15.4)	70 (24.6)
**Mechanism of Injury**	700		
Sports/Recreation		270 (65.1)	207 (72.6)
Non-sport/Fall		138 (33.3)	70 (24.6)
Motor vehicle collision		4 (1.0)	3 (1.1)
Assault		3 (0.7)	5 (1.8)
**BESS tandem stance, mean (SD), No. errors**	700	3.42 (3.41)	4.25 (3.73)

© 2023 Vaughan CG et al. *JAMA Network Open.*

Variable	*N*	Early RTS: 0-2 missed school days (n=415) No. (%)	Late RTS: 3+ missed school days (n=285) No. (%)
**Amnesia following injury**	700	125 (30.1)	98 (34.4)
**SAC orientation, mean (SD), raw**	700	3.64 (0.67)	3.54 (0.78)
**SAC immediate memory, mean (SD), raw**	700	13.57 (1.48)	13.32 (1.92)
**SAC concentration, mean (SD), raw**	700	4.01 (1.18)	3.95 (1.21)
**SAC delayed recall, mean (SD), raw**	700	4.19 (1.12)	3.95 (1.20)
**SAC total score, mean (SD), normalized**	700	0.16 (1.07)	-0.10 (1.32)
**PCSI-P Headache, mean (SD), delta score**	700	3.18 (1.89)	3.89 (1.82)
**PCSI-P Nausea, mean (SD), delta score**	700	1.92 (2.12)	2.38 (2.17)
**PCSI-P Balance, mean (SD), delta score**	700	1.24 (1.75)	1.62 (1.95)
**PCSI-P Dizziness, mean (SD), delta score**	700	2.34 (2.00)	2.94 (2.09)
**PCSI-P Drowsy, mean (SD), delta score**	700	2.47 (2.06)	3.05 (2.10)
**PCSI-P Sleep more, mean (SD), delta score**	700	1.07 (1.90)	1.56 (2.19)
**PCSI-P Sensitivity to light, mean (SD), delta score**	700	0.95 (1.71)	1.71 (2.04)
**PCSI-P Sensitivity to noise, mean (SD), delta score**	700	0.81 (1.54)	1.32 (1.84)
**PCSI-P Irritability, mean (SD), delta score**	700	0.54 (1.23)	0.95 (1.64)
**PCSI-P Sad, mean (SD), delta score**	700	1.05 (1.70)	1.39 (1.87)
**PCSI-P Nervous, mean (SD), delta score**	700	0.65 (1.36)	0.85 (1.49)
**PCSI-P More emotional, mean (SD), delta score**	700	1.04 (1.69)	1.48 (1.95)
**PCSI-P Mental fog, mean (SD), delta score**	700	1.50 (1.85)	2.11 (2.05)
**PCSI-P Difficulty concentrating, mean (SD), delta score**	700	0.88 (1.49)	1.21 (1.77)
**PCSI-P Difficulty remembering, mean (SD), delta score**	700	0.72 (1.52)	1.14 (1.78)
**PCSI-P Vision, mean (SD), delta score**	700	1.08 (1.80)	1.27 (1.86)
**PCSI-P Fatigue, mean (SD), delta score**	700	2.44 (2.03)	2.94 (1.99)
**PCSI-P Confuse, mean (SD), delta score**	700	0.57 (1.31)	0.88 (1.62)
**PCSI-P Clumsy, mean (SD), delta score**	700	0.65 (1.36)	1.01 (1.69)
**PCSI-P Answers slowly, mean (SD), delta score**	700	1.16 (1.62)	1.67 (1.91)
**PCSI-C at ED visit, mean (SD), total score**	700	9.91 (5.57)	12.83 (6.16)
**Day of initial injury**	700		
Sunday		52 (12.5)	61 (21.4)
Monday		51 (12.3)	56 (19.6)
Tuesday		60 (14.5)	47 (16.5)
Wednesday		67 (16.1)	40 (14.0)

© 2023 Vaughan CG et al. *JAMA Network Open.*

Variable	N	Early RTS: 0-2 missed school days (n=415)<br>No. (%)	Late RTS: 3+ missed school days (n=285)<br>No. (%)
Thursday		78 (18.8)	29 (10.2)
Friday		56 (13.5)	19 (6.7)
Saturday		51 (12.3)	33 (11.6)

© 2023 Vaughan CG et al. *JAMA Network Open.*

eTable 3. Descriptive statistics of Key Variables Applied in Propensity Score Analysis for Each Age Group, Stratified by Early vs Late Return-to-School for Participants Ages 13 to 18 Years

Variable	*N*	Early RTS: 0-2 missed school days (n=264) No. (%)	Late RTS: 3+ missed school days (n=383) No. (%)
**Age, mean (SD), years**	647	15.02 (1.23)	15.12 (1.25)
**Sex**	647		
Male		156 (59.1)	197 (51.4)
Female		108 (40.9)	186 (48.6)
**Maximum symptom duration from previous concussion(s) (weeks)**	647		
Never had concussion		181 (68.6)	244 (63.7)
<1 week		32 (12.1)	44 (11.5)
1-2 weeks		24 (9.1)	27 (7.0)
3-4 weeks		8 (3.0)	32 (8.4)
5-8 weeks		5 (1.9)	14 (3.7)
>8 weeks		14 (5.3)	22 (5.7)
**Time between head injury and triage, mean (SD), hours**	647	8.54 (11.82)	12.70 (13.84)
**Prior treatment for headache**	647	46 (17.4)	78 (20.4)
**Personal history of migraine**	647	37 (14.0)	69 (18.0)
**Family history of migraine**	647		
No		132 (50.0)	205 (53.5)
Yes		125 (47.3)	171 (44.6)
Unknown		7 (2.7)	7 (1.8)
**History of learning disability**	647	19 (7.2)	34 (8.9)
**History of attention deficit disorder**	647	24 (9.1)	32 (8.4)
**History of other developmental disorders**	647	10 (3.8)	11 (2.9)
**History of anxiety**	647	19 (7.2)	54 (14.1)
**Seizure following injury**	647	4 (1.5)	5 (1.3)
**ACE - Appears dazed and confused**	647	131 (49.6)	222 (58.0)
**ACE - Appears confused about events**	647	59 (22.3)	122 (31.9)
**ACE - Answers questions slowly**	647	109 (41.3)	166 (43.3)
**ACE - Repeats questions**	647	36 (13.6)	63 (16.4)
**ACE - Forgetful of recent information**	647	58 (22.0)	103 (26.9)
**Mechanism of Injury**	647		
Sports/Recreation		210 (79.5)	310 (80.9)
Non-sport/Fall		41 (15.5)	62 (16.2)
Motor vehicle collision		9 (3.4)	3 (0.8)
Assault		4 (1.5)	7 (1.8)
Other		0 (0.0)	1 (0.3)

© 2023 Vaughan CG et al. *JAMA Network Open.*

Variable	*N*	Early RTS: 0-2 missed school days (n=264) No. (%)	Late RTS: 3+ missed school days (n=383) No. (%)
BESS tandem stance, mean (SD). No. errors	647	3.40 (3.63)	3.78 (3.87)
Amnesia following injury	647	70 (26.5)	133 (34.7)
SAC orientation, mean (SD), raw score	647	3.80 (0.43)	3.73 (0.55)
SAC immediate memory, mean (SD), raw score	647	13.84 (1.74)	13.76 (1.50)
SAC concentration, mean (SD), raw score	647	4.56 (1.20)	4.46 (1.20)
SAC delayed recall, mean (SD), raw score	647	4.04 (1.17)	4.09 (1.21)
SAC total, mean (SD), normalized score	647	-0.27 (1.56)	-0.39 (1.55)
PCSI-P Headache, mean (SD), delta score	647	3.32 (1.85)	3.80 (1.77)
PCSI-P Nausea, mean (SD), delta score	647	1.73 (2.07)	2.34 (2.12)
PCSI-P Balance, mean (SD), delta score	647	1.36 (1.77)	1.70 (1.98)
PCSI-P Dizziness, mean (SD), delta score	647	2.42 (1.99)	2.75 (1.98)
PCSI-P Drowsy, mean (SD), delta score	647	2.21 (2.08)	3.00 (2.11)
PCSI-P Sleep more, mean (SD), delta score	647	0.84 (1.76)	1.56 (2.10)
PCSI-P Sensitivity to light, mean (SD), delta score	647	1.45 (2.05)	2.22 (2.26)
PCSI-P Sensitivity to noise, mean (SD), delta score	647	1.31 (1.93)	1.99 (2.12)
PCSI-P Irritability, mean (SD), delta score	647	0.66 (1.50)	0.98 (1.68)
PCSI-P Sad, mean (SD), delta score	647	0.85 (1.53)	1.11 (1.81)
PCSI-P Nervous, mean (SD), delta score	647	0.47 (1.21)	0.68 (1.45)
PCSI-P More emotional, mean (SD), delta score	647	0.82 (1.56)	1.22 (1.88)
PCSI-P Mental fog, mean (SD), delta score	647	1.54 (1.90)	2.16 (2.03)
PCSI-P Difficulty concentrating, mean (SD), delta score	647	1.00 (1.54)	1.60 (1.94)
PCSI-P Difficulty remembering, mean (SD), delta score	647	0.88 (1.62)	1.31 (1.96)
PCSI-P Vision, mean (SD), delta score	647	1.14 (1.78)	1.42 (1.98)
PCSI-P Fatigue, mean (SD), delta score	647	2.04 (2.09)	3.08 (2.00)
PCSI-P Confuse, mean (SD), delta score	647	0.57 (1.29)	0.87 (1.56)
PCSI-P Clumsy, mean (SD), delta score	647	0.67 (1.36)	0.85 (1.53)
PCSI-P Answers slowly, mean (SD), delta score	647	1.24 (1.70)	1.54 (1.84)
PCSI-C at ED visit, mean (SD), total score	647	28.26 (19.82)	39.34 (21.16)
Day of initial injury	647		
Sunday		36 (13.6)	70 (18.3)
Monday		31 (11.7)	79 (20.6)

© 2023 Vaughan CG et al. *JAMA Network Open.*

Variable	*N*	Early RTS: 0-2 missed school days (n=264)<br>No. (%)	Late RTS: 3+ missed school days (n=383)<br>No. (%)
Tuesday		31 (11.7)	52 (13.6)
Wednesday		32 (12.1)	53 (13.8)
Thursday		48 (18.2)	39 (10.2)
Friday		39 (14.8)	36 (9.4)
Saturday		47 (17.8)	54 (14.1)

© 2023 Vaughan CG et al. *JAMA Network Open.*

eFigure 1. Standardized Mean Differences Before and After Applying IPTW (Age 5−7)

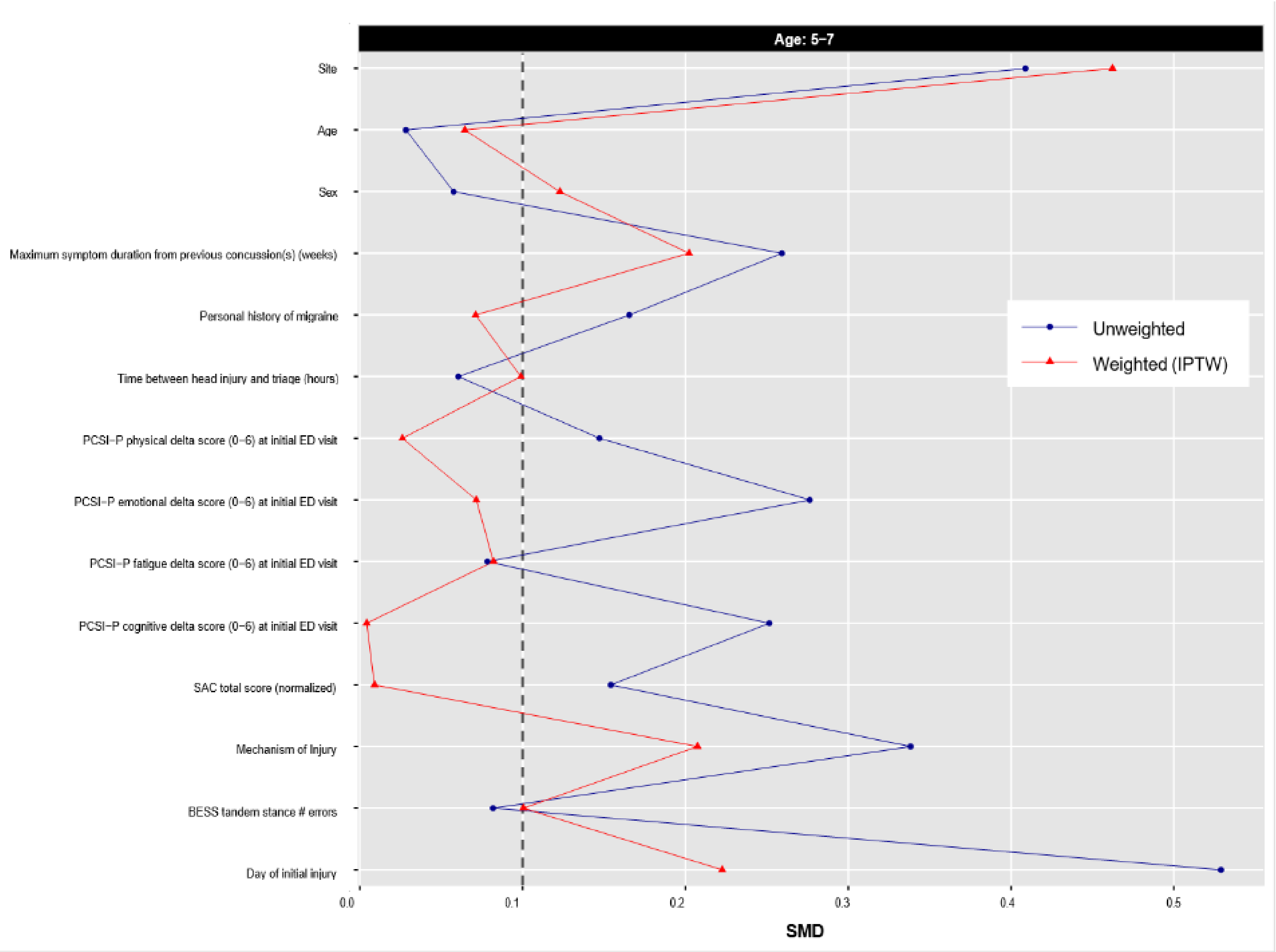

© 2023 Vaughan CG et al. *JAMA Network Open.*

eFigure 2. Standardized Mean Differences Before and After Applying IPTW (Age 8-12 Years and Age 13-18 Years)

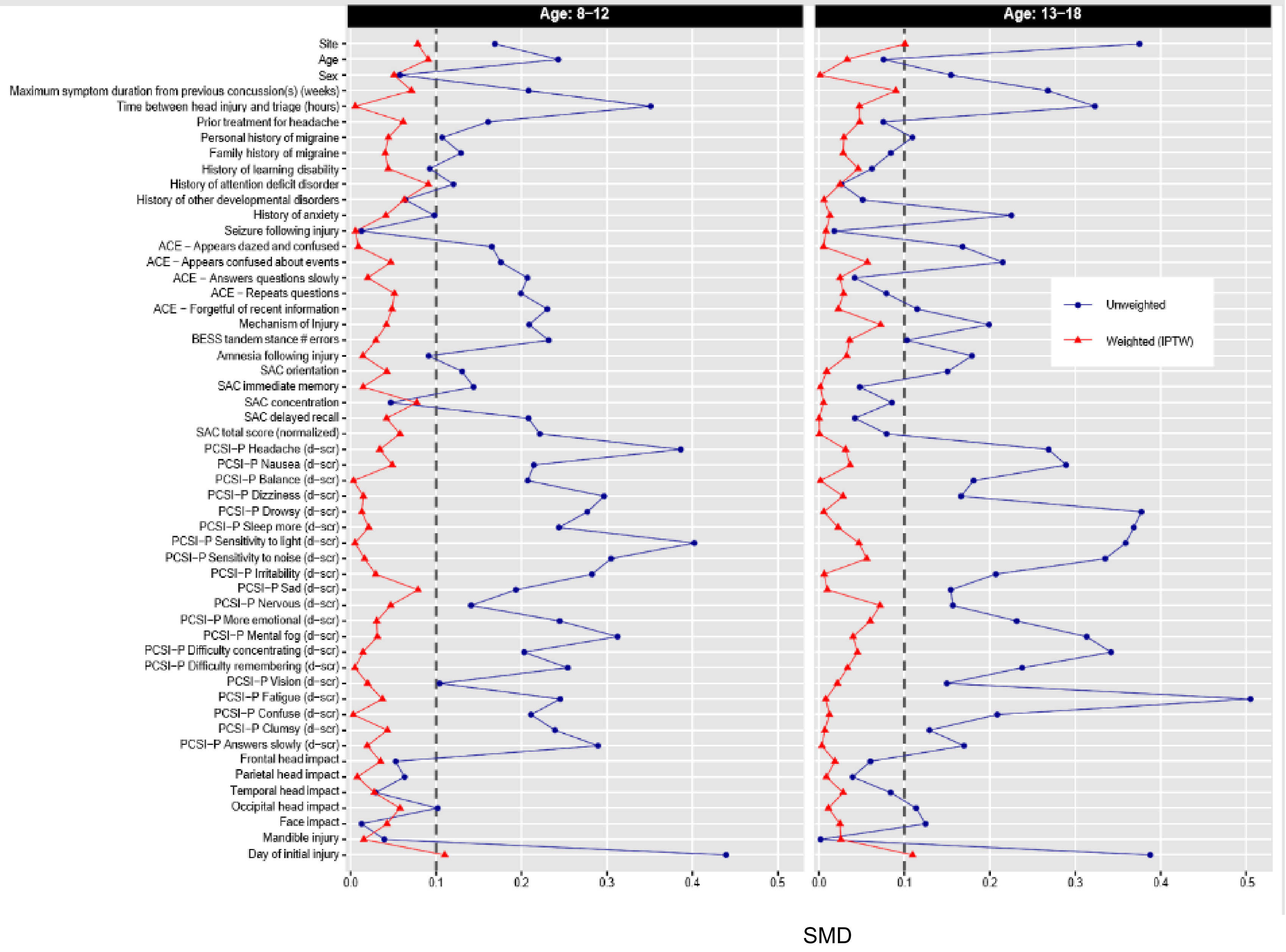

© 2023 Vaughan CG et al. *JAMA Network Open.*

eFigure 3. PCSI Summed Score at 2 Week Follow–up (Primary Study Outcome), Stratified by Early or Late Return-To-School And Age Group

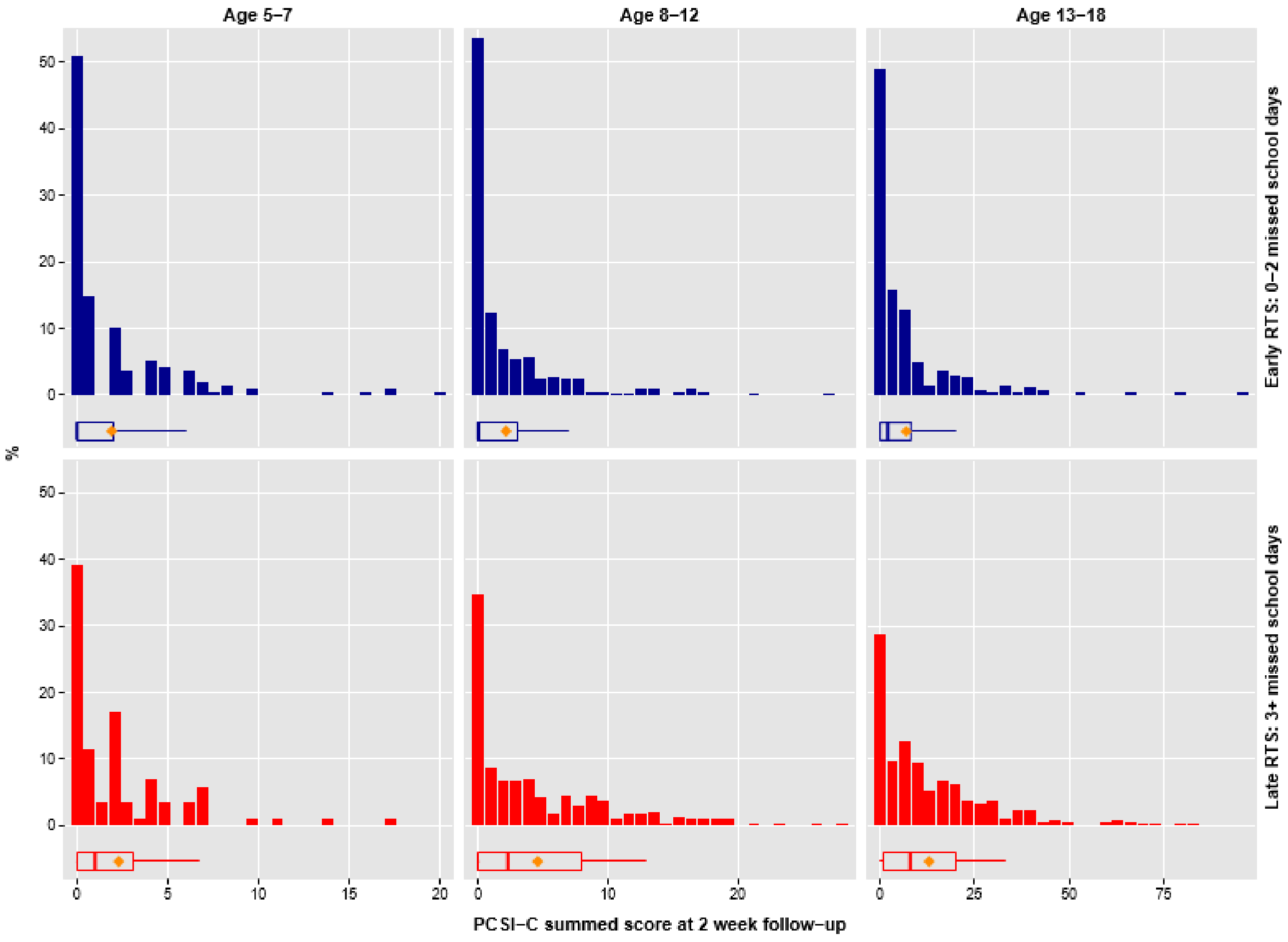

© 2023 Vaughan CG et al. *JAMA Network Open.*

© 2023 Vaughan CG et al. *JAMA Network Open.*
