# Supplementary material for: Association Between Early Return to School Following Acute Concussion and Symptom Burden at 2 Weeks Postinjury
Source: JAMA Netw Open. 2023 Jan 20;6(1):e2251839. doi: 10.1001/jamanetworkopen.2022.51839 (PMC9860528; doi:10.1001/jamanetworkopen.2022.51839)
Supplement: Supplement 2. — Nonauthor Collaborators [file jamanetwopen-e2251839-s002.pdf]

Supplemental Online Content: Nonauthor Collaborators
*First name, last name, and suffix (if applicable) are required and will appear in PubMed.

*Group Name(s): Perc 5P Concussion Team							
***First Name and Middle Initial(s)**	***Last Name**	***Suffix (eg, Jr, III)**	Academic Degrees	Institution	Location (city, state/province, country)	Role or Contribution, eg, chair, principal investigator	Group (if more than 1 Group listed in the byline) and/or Subgroup (eg, Steering Committee)
Candice	McGahern		BA	Children's Hospital of Eastern Ontario	Ottawa, Ontario, Canada	National Coordinator	
Angelo	Mikrogianakis		MD	Alberta's Children Hospital	Calgary, Alberta, Canada	Site lead	
Ken	Farion		MD	Children's Hospital of Eastern Ontario	Ottawa, Ontario, Canada	Site lead	
Karen	Barlow		MD	Alberta's Children Hospital	Calgary, Alberta, Canada	Co-investigator	
Alexander S	Dubrovsky		MDCM, MSc	Montreal Children's Hospital	Montreal, Quebec, Canada	Co-investigator	
Willem	Meeuwisse		MD, PhD	University of Calgary	Calgary, Alberta, Canada	Co-investigator	
William	Meehan	III	MD	Boston Children's Hospital	Boston, Massachusetts, US	Co-investigator	
Yael	Kamil		BSc	Children's Hospital of Eastern Ontario	Ottawa, Ontario, Canada	Data Manager	
Miriam	Beauchamp		PhD	Sainte-Justine Hospital	Montreal, Quebec, Canada	Neuropsychologist site lead and co-Investigator	
Blaine	Hoshizaki		PhD	University of Ottawa	Ottawa, Ontario, Canada	Co-investigator	
Peter	Anderson		PhD	Children's Hospital of Eastern Ontario	Ottawa, Ontario, Canada	Neuropsychologist site lead and co-Investigator	
Brian L.	Brooks		PhD	Alberta's Children Hospital	Calgary, Alberta, Canada	Neuropsychologist site lead and co-Investigator	
Michael	Vassilyadi		MDCM, MSc	Children's Hospital of Eastern Ontario	Ottawa, Ontario, Canada	Co-investigator	
Terry	Klassen		MD	Manitoba Children's Hospital	Winnipeg, Manitoba, Canada	Co-investigator	

Supplemental Online Content: Nonauthor Collaborators
*First name, last name, and suffix (if applicable) are required and will appear in PubMed.

***First Name and Middle Initial(s)**	***Last Name**	***Suffix (eg, Jr, III)**	Academic Degrees	Institution	Location (city, state/province, country)	Role or Contribution, eg, chair, principal investigator	Group (if more than 1 Group listed in the byline) and/or Subgroup (eg, Steering Committee)
Michelle	Keightley		MD	Bloorview Research Institute	Toronto, Ontario, Canada	Neuropsychologist site lead, co-Investigator	
Lawrence	Richer		MD	Stollery Children's Hospital	Edmonton, Alberta, Canada	Co-investigator	
Carol	Dematteo		MSc	McMaster University	Hamilton, Ontario, Canada	Xo-investigator	
Nick	Barrowman		PhD	Children's Hospital of Eastern Ontario	Ottawa, Ontario, Canada	Biostatistician	
Mary	Aglipay		MSc	Children's Hospital of Eastern Ontario	Ottawa, Ontario, Canada	Biostatistician	
Anne	Grool		MD, PhD	Children's Hospital of Eastern Ontario	Ottawa, Ontario, Canada	Post-Doctoral Fellow	
