# Supplementary material for: Association Between Early Return to School Following Acute Concussion and Symptom Burden at 2 Weeks Postinjury
Source: JAMA Netw Open. 2023 Jan 20;6(1):e2251839. doi: 10.1001/jamanetworkopen.2022.51839 (PMC9860528; doi:10.1001/jamanetworkopen.2022.51839)
Supplement: Supplement 3. — Data Sharing Statement [file jamanetwopen-e2251839-s003.pdf]

Data Sharing Statement

Vaughan. Association Between Early Return to School Following Acute Concussion and Symptom Burden at 2 Weeks Postinjury. *JAMA Netw Open*. Published January 20, 2023. doi:10.1001/jamanetworkopen.2022.51839

Data
Data available: No
